# A facile template route to periodic mesoporous organosilicas nanospheres with tubular structure by using compressed CO_2_

**DOI:** 10.1038/srep45055

**Published:** 2017-03-20

**Authors:** Xin Huang, Wei Li, Meijin Wang, Xiuniang Tan, Qian Wang, Cheng Wang, Mengnan Zhang, Jing Yuan

**Affiliations:** 1 Department of Chemistry, Capital Normal University, Beijing, 100048, China

## Abstract

Periodic mesoporous organosilicas (PMOs) nanospheres with tubular structure were prepared with compressed CO_2_ using cationic and anionic mixed surfactant (CTAB/SDS) and triblock copolymer Pluronic P123 as bi-templates. TEM, N_2_ adsorption-desorption, solid NMR, and FTIR were employed to characterize the obtained materials. Compressed CO_2_ severed as acidic reagent to promote the hydrolysis of organosilicas, and could tune the morphology and structure of the obtained PMOs nanomaterials simple by adjusting the CO_2_ pressure during the synthesis process. Rhodamine B (RB) and Ibuprofen (IBU), as the model dye and drug, were loaded into the prepared nanomaterials to reveal its adsorption and desorption ability. Furthermore, different molars of the surfactant (CTAB/SDS) and organosilane precursor (BTEB) were investigated to show the effect of the surfactant concentration on the morphology and structure of the PMOs prepared with compressed CO_2_, and some different structures were obtained. A possible mechanism for the synthesis of PMOs with tubular structure using compressed CO_2_ was proposed based on the experimental results.

Hybrid materials with organic group distributed in the inorganic framework have attracted many attentions because they combine the functional versatility of organics with the advantages of thermal stability of inorganic substrates^1^,^2^,^3^,^4^,^5^,^6^,^7^,^8^,^9^,^10^. Periodic mesoporous organosilicas (PMOs) are one of the most representative organic-inorganic mesoporous materials with the organic components homogeneously distributed over the whole inorganic frameworks at the molecular level^5^,^11^,^12^,^13^. Because of their high surface areas, mesoscale porous structures enabling easy molecular diffusion, diversity of framework components, and light-harvesting properties of the frameworks^14^,^15^, PMOs are promising for various functional applications such as adsorbents^16^, solid catalysts^17^, luminescent materials^18^,^19^, and nano-vessels for biological applications^20^. The catalytic activity of PMOs is determined by their morphological characteristics and pore structure, which affect the number of active sites, reactant or product diffusion, and accessibility of the active sites to reactants^21^. Therefore, these PMOs with open and accessible mesopore channels, especially for the tubular structure, facilitated access of the guest molecules to the internal surface of the mesostructure to realize synergistic effects^22^,^23^,^24^,^25^,^26^,^27^,^28^,^29^,^30^,^31^,^32^,^33^,^34^,^35^,^36^,^37^,^38^,^39^,^40^,^41^,^42^,^43^. In addition, PMOs with tubular structure are preferable in some special applications, such as drug delivery or adsorption of waste water, because these particles could be selectively accumulated by tissue for their enhanced permeability and retention^27^,^44^,^45^,^46^,^47^,^48^,^49^,^50^,^51^,^52^,^53^,^54^,^55^,^56^,^57^. However, not many synthesis approaches have been successfully developed to fabricate PMOs with tubular structures for the poor control in pore structure and particle morphology^58^,^59^,^60^. Recently bifunctional PMOs with tunable mesoporous size were successfully synthesized through a simple and effective approach using compressed CO_2_ to tune the acidity and template structure^61^. It is a universal method to provide unlimited potential and possibility for synthesizing different kinds of PMOs materials. Therefore, the PMOs with tubular structure were prepared via this green and facile approach in this study. Three different kinds of surfactants, cationic surfactant (CTAB), anionic surfactant (SDS) and nonion surfactant (triblock copolymer Pluronic P123), were employed as soft template in the synthesis. Therein, CTAB and SDS were self-assembly for their strong electrostatic interactions to form a micelles or vesicle structure at different concentrations and molar ratio while P123 offers the mesopores on the shell via a templating-assembly approach^62^. Thus, the mixed surfactant (CTAB/SDS-P123) system and 1,4-bis(triethoxysilyl)benzene (BTEB) were served as bi-templates and the organosilica precursor in the synthesis of PMOs respectively. Notably, channel on the PMOs nanospheres’ shell were clearly observed by TEM images.

To further investigate the adsorption and desorption ability of the obtained PMOs, dye Rhodamine B (RB) and drug ibuprofen (IBU) were employed in the study. Rhodamine B (RB), a synthetically prepared carcinogenic xanthine dye widely used in industries, has caused serious industrial wastewater pollution^63^,^64^. Therefore, many different substances have been investigated to eliminate RB pollution in waste water. PMOs were demonstrated highly effective adsorption to RB^65^,^66^. In this study, the prepared PMOs nanomaterials were employed to absorb RB in the water using the UV/visible spectrophotometer. In light of its potential application in the area of drug delivery, ibuprofen (IBU), a typical anti-inflammatory drug was chosen for assessing the adsorption ability of the PMOs nanospheres. Furthermore, different molar ratios of the surfactants and organosilica precursor in the synthesis of PMOs nanomaterials have also been investigated to get a detailed understanding of the role that surfactants played in the structure formation by using the compressed CO_2_.

## Result and Discussion

### Structural characterization

**Figure 1:**
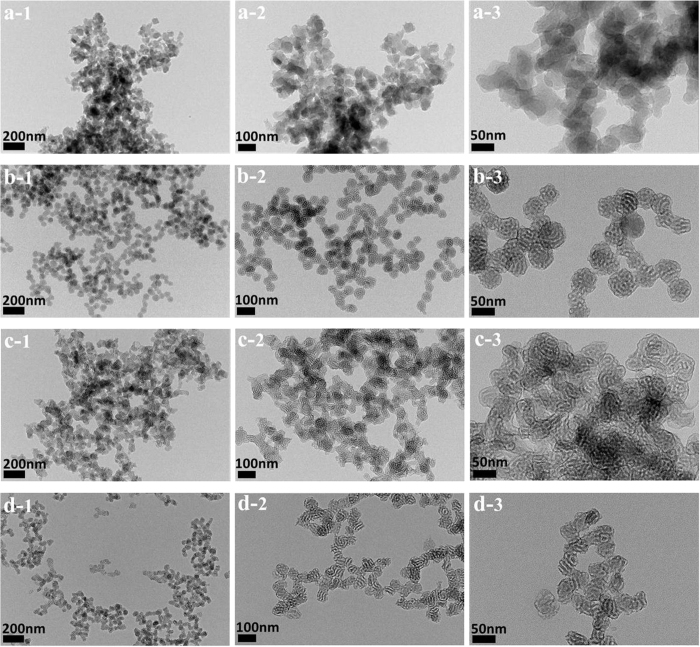
TEM images of the PMOs nanospheres with tubular structure synthesized using CTAB/SDS and P123 as bi-templates at the CO_2_ pressure of (**a**) 2.90, (**b**) 3.90, (**c**) 4.90, and (**d**) 5.90 MPa.

No precipitation was observed when the organosilica precursors BTEB was hydrolyzed in CTAB/SDS and P123 bi-template surfactant solutions without addition of liquid acid or compressed CO_2_. White precipitates were formed when CO_2_ was charged into the autoclave with a suitable pressure for 24 hours. Figure 1 shows the TEM imagines of the obtained materials synthesized at different CO_2_ pressures. Uniform and tubular structure was clearly observed in the samples prepared at 3.90, 4.90 and 5.90 MPa. The nanoparticles presented spheres morphology with the size in the range of 50–60 nm when the pressure of CO_2_ was 3.90 MPa (Fig. 1b). When the pressure increased to 4.90 MPa, the nanospheres gathered together and gradually formed tubular strcutures with worm-like mesopores (Fig. 1c). While at the pressure of 5.90 MPa, the obtained nanomaterials presented a clear worm-like morphology in the TEM (Fig. 1d) with highly uniform pore size. However, the tubular structure could not be observed when the pressure decreased to 2.90 MPa (Fig. 1a). It may be due to the low pressure of compressed CO_2_ cannot provide enough acidity during the synthesis process of PMOs^39^. We also noticed that the obtained nanoparticles are all presented an aggregation in the TEM images. It may be attributed to the surfactant aggregation during the self-assembly process, which served as the soft template to promote the further growth of these mesoporous materials, similar to previous research through a soft-template method with normal catalysts (such as acid or other additions)^2^,^67^.

**Figure 2:**
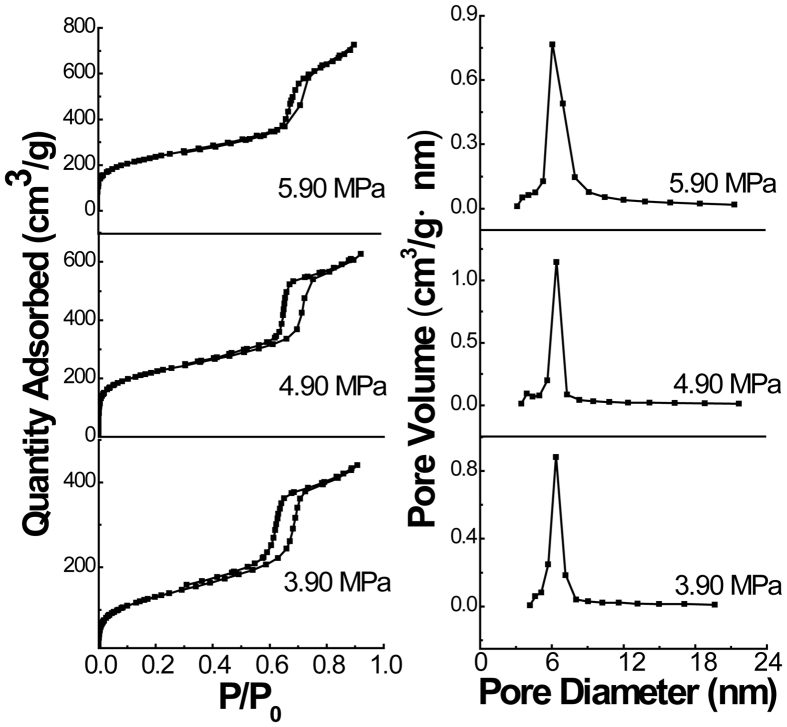
Nitrogen adsorption–desorption isotherms and pore size distributions of the PMOs nanospheres with tubular structure synthesized at different CO_2_ pressures.

N_2_ adsorption-desorption isotherms and the corresponding BJH pore size distributions of the obtained nanomaterials with tubular structure are shown in Fig. 2. The surface areas were calculated by the BET method, and the pore size distributions were evaluated using the adsorption branches of the sorption isotherms with the BJH method, which are listed in Table 1. All the isotherms are of type IV according to IUPAC classification and exhibit H1 hysteresis loops, which is typical for mesoporous materials with cylindrical channels^68^. The pore size distribution indicates the existence of mesopores with an average size of 5.7 nm according to the BJH formula at the prepared pressure of 3.90 MPa. While the pore volume and BET-specific surface area were calculated to be 0.68 cm^3^/g and 477 m^2^/g. When the pressure rose to 4.90 MPa, the corresponding pore size and pore volume also increased to 6.4 nm and 0.97 cm^3^/g, respectively. Meanwhile, the BET-specific surface area increased to 781 m^2^/g. Similarly, the pore size, pore volume and the surface area also increased to 6.6 nm, 1.11 cm^3^/g and 815 m^2^/g with increasing pressure to 5.90 MPa. Evidently, the pore size calculated by the sorption isotherms with BJH method is roughly corresponding with that estimated from TEM images. Furthermore, the pore size, pore volume and surface area of obtained PMOs increased monotonically with increasing pressure of CO_2_. The increased pore size, pore volume and the surface area will benefit a number of applications such as protein encapsulation and drug delivery.

### Compositional information

**Figure 3:**
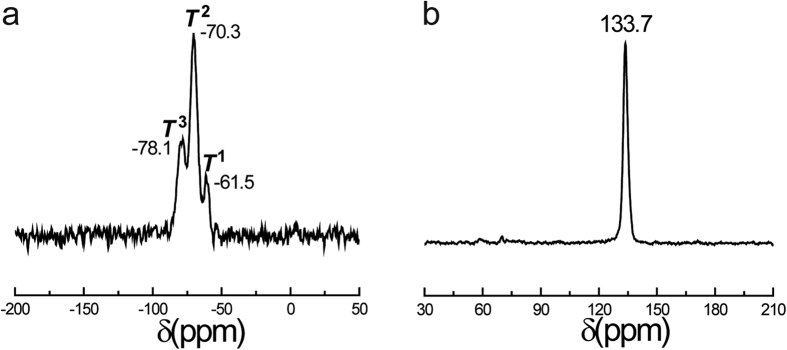
(**a**) ^13^Si MAS and (**b**) ^13^C CP MAS NMR spectra of PMOs synthesized with CTAB/SDS and P123 as bi-templates at 4.90 MPa of CO_2_ pressure.

NMR characterization was performed to verify the composition of the covalently bonded organic bridging groups in the PMOs materials. The PMOs sample prepared at 4.90 MPa was chosen to confirm the component since organosilicate structure was almost unchanged with different CO_2_ pressures during the synthesis^61^. Solid-state ^29^Si magic-angle spinning (MAS) showed characteristic *T*^n^ signals attributed to [C–Si(OSi)(OH)_2_] (*T*^1^ at −61.5 ppm), [C–Si(OSi)_2_(OH)] (*T*^2^ at −70.3 ppm) and [C–Si(OSi)_3_] (*T*^3^ at −78.1 ppm) (Fig. 3a), and virtually no *Q*^n^ signals (Si sites attached to four oxygen atom) between −98 and −111 ppm. This confirmed the existence of organic group in the sample and showed that Si–C bond keep uncleaved during the synthesis and surfactant extraction process^69^. Solid-state ^13^C cross-polarization (CP) MAS NMR, as shown in Fig. 3b, is dominated by a single resonance at 133.7 ppm, corresponding to the attachment between aryl carbon and silicon atoms^68^, which confirms the aromatic functional group has bridged into the mesoporous materials. Signal peak at about 70 ppm attributed to the carbon of surfactant P123 is hardly observed, suggesting that all P123 has been removed through solvent extraction method^70^.

**Figure 4:**
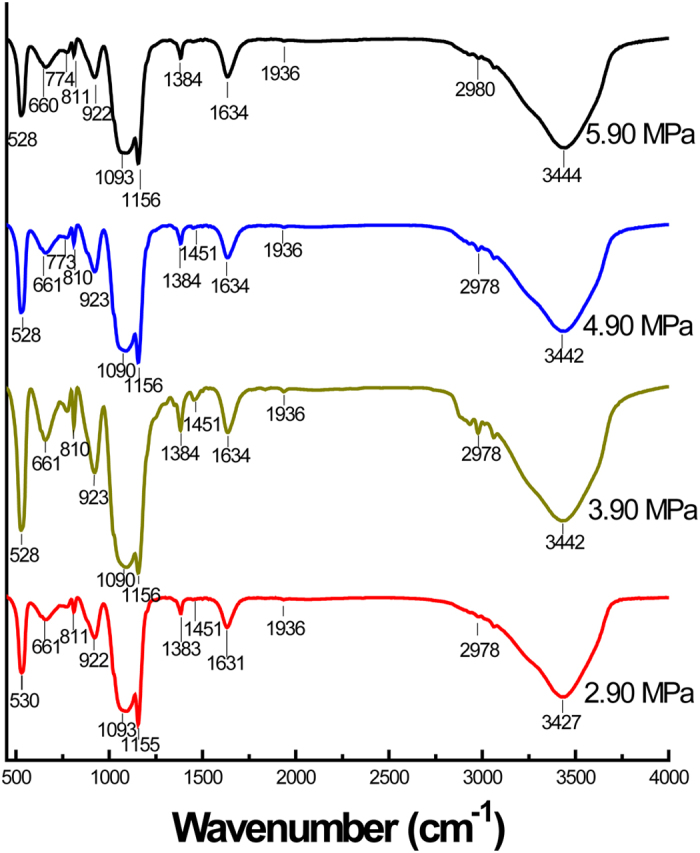
FTIR spectra of PMOs nanospheres synthesized with different CO_2_ pressures.

FTIR spectroscopy was implemented to characterize the frame structure of the materials and shown in Fig. 4. The strong bands at around 3427 cm^−1^ is attributed to the stretching and deformational vibrations of the residual water^71^. The asymmetrical stretching of Si–O–Si at about 1090 cm^−1^ and the symmetric stretching vibration of Si–O at about 810 cm^−1^ indicate the formation of siloxanes network in the framework of all PMOs samples. The C–H out-of-plane flexural vibration at about 774 cm^−1^ and the overtone band in the range from 2100 cm^−1^ to 1600 cm^−1^ are generated by the C–H vibration of monosubstituted benzene. All of these peaks can be detected in the prepared PMOs nanomaterials, which indicate that benzene group has been successfully loaded in these samples. Moreover, the aromatic ring vibrations at about 1450 cm^−1^ and 1634 cm^−1^ are clearly observed in the spectrum, proving the existence of benzene groups bridging to the framework with covalent bond in the prepared materials. It is obvious that all FTIR spectroscopy are almost same, further indicating that the organosilicate structure of PMOs is stable and no damage during the syntheses under different CO_2_ pressures.

### Adsorption application of the prepared PMOs

**Figure 5:**
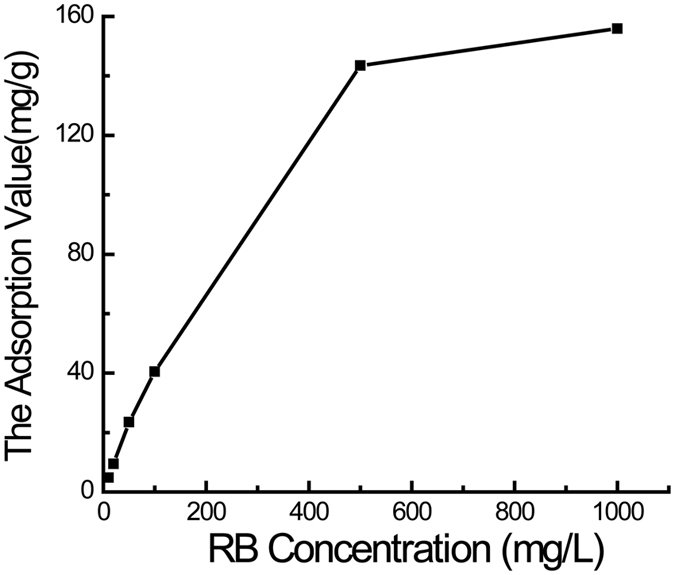
Plot of RB adsorption capacity of PMOs nanospheres in different initial RB concentrations.

One of the most important applications of PMOs with tubular structure was the adsorption feature. Dyes are primary water pollutants generally in the effluents of the textile, leather, food processing, dyeing, cosmetics, paper, and dye manufacturing industries^69^. The investigation of the dye adsorption capacity was undertaken by selecting a typical dye, RB, as a model molecule. It has been reported that the hydrogen bonds were the main interactions of RB dye molecules with the phenyl-bridged PMOs matrices^65^. PMOs prepared at 4.90 MPa were chosen to measure the adsorption capacity and the corresponding adsorption equilibrium isotherm is shown in Fig. 5. As can be seen from the result, the adsorption capacity of the prepared PMOs nanomaterials increased with the increase of initial RB concentrations. According to Fig. 5, the adsorption value of RB increased significantly from 4.8 to 153.2 mg/g with the increase of the initial RB concentration in the range of 10–1000 mg/L. Furthermore, the adsorption capacity of the prepared PMOs obviously far exceed that of the other PMOs, especially when the initial RB solution is lower than 500 mg/L, which increased from about 10 mg/g to only 50 mg/g with the increase of the initial RB concentration^65^. It was the high specific surface area and the special tubular structure that provide excellent adsorption capacity for the prepared PMOs nanomaterials. To further investigate the adsorption of the RB molecules, N_2_ adsorption-desorption isotherms and structure properties of the PMOs after loading RB has been conducted and shown in the supporting material (Figure S1 and Table S1). Just as shown in the figure, the surface area of the PMOs synthesized at 4.90 MPa decreases from 781 to 601 m^2^/g after loading RB. Meanwhile, the corresponding pore size and pore volume decrease from 6.4 nm and 0.97 cm^3^/g to 5.4 nm and 0.71 cm^3^/g respectively. The results proved that the RB molecules have been adsorbed in the pores of the PMOs.

**Figure 6:**
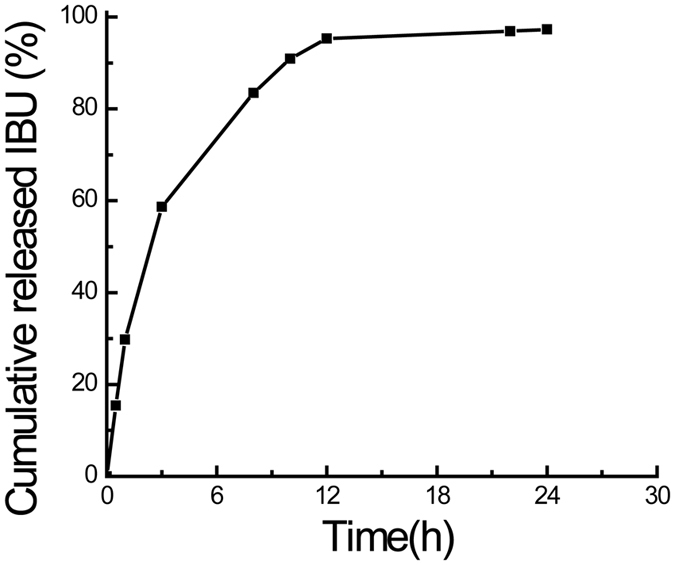
Release of IBU from the prepared PMOs nanospheres synthesized at 4.90 MPa.

PMOs have recently been explored as effective drug delivery carriers to fight against various kinds of diseases because of the ordered mesopores structure and organic groups^72^. To further develop the potential applications of the prepared PMOs with tubular structure in biomedical fields, the adsorption and desorption capacity was assessed with a typical anti-inflammatory drug IBU (Fig. 6). The loading amount of IBU was measured to be 317.3 mg (IBU)/g of SiO_2_ onto the prepared PMOs nanospheres. The result was almost same with that of SBA-15^66^. Obviously, there is a burst release of IBU (90.9% of the loaded amount) in the first 10 hours, which reached approximately 97.3% in 24 h. The initial burst release may be due to the excessive IBU molecules which were weakly entrapped inside the mesopores or on the outer surface of PMOs, whereas the slow release of the rest of the IBU from PMOs is attributed to the π-π stacking interactions between IBU and the functionalized mesoporous surface^73^.

### PMOs nanomaterials synthesized with different molars ratios of CTAB/SDS and BTEB

**Figure 7:**
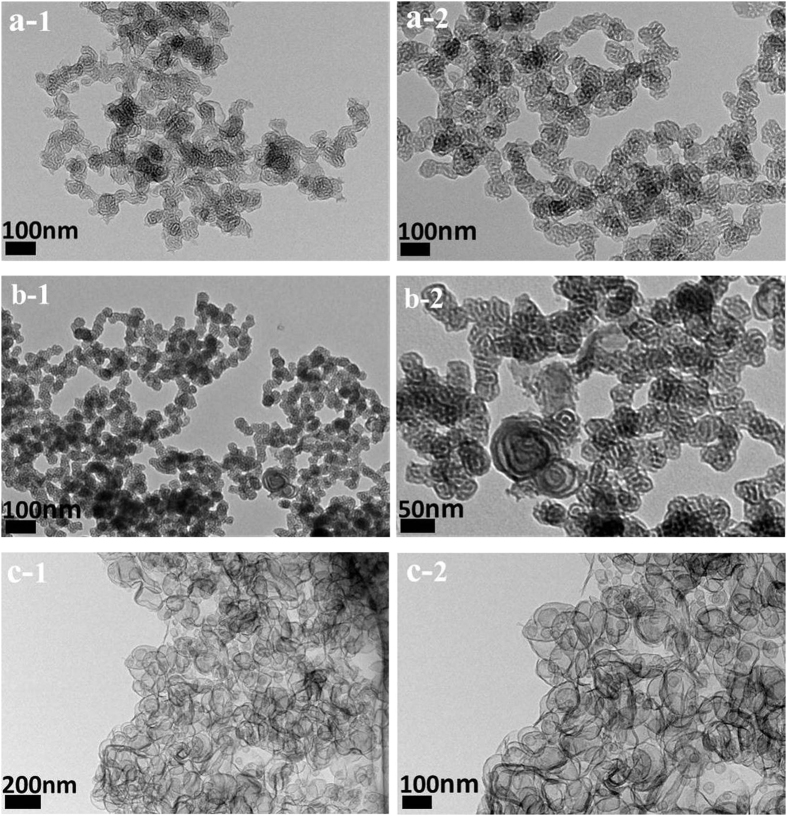
TEM images of the PMOs synthesized with surfactants (CTAB/SDS)/P123 and BTEB at different molar ratio (**a**) 0.004:1, (**b**) 0.017:1, and (**c**) 0.068:1 with the CO_2_ pressure of 4.90 MPa.

To further investigate the effect of the surfactant concentration on the morphology and structure of the PMOs prepared with compressed CO_2_, different molar ratios of the total surfactant (CTAB/SDS) and organosilane precursor (BTEB) were selected to synthesis PMOs nanomaterials. Figure 7 shows the TEM images of PMOs synthesized at different molar ratios of surfactants and BTEB. Just as mentioned above, PMOs nanomateials presented worm-like nanoparticles when the molar ratio of the surfactant and organosilane precursors is 0.004:1 with the CO_2_ pressure of 4.90 MPa (Fig. 7a). However, the morphology of PMOs is predominated by hollow nanospheres (Fig. 7c) when the molar ratio increased to 0.068:1 with the same CO_2_ pressure. It is very interesting to note that the worm-like nanoparticles and hollow nanospheres coexisted in the sample when the molar ratio is 0.017:1, indicating that the worm micelles and spherical vesicles can be simultaneously obtained in the solution under this condition (Fig. 7b). It was due to the surfactant self-aggregations varied with different surfactant concentrations in the solution, as we have already known, the mixture of CTAB and SDS in aqueous solution possesses a variety of microstructure formed by self-assembly^64^.

### Formation mechanism of PMOs with tubular structure by using compressed CO_2_

**Figure 8:**
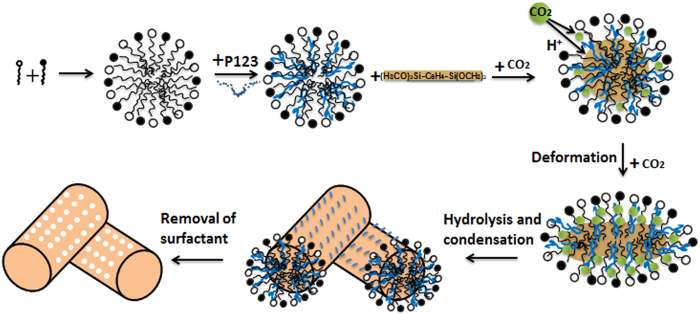
Schematic illustration of formation of PMOs nanospheres with tubular structure synthesized using CTAB/SDS and P123 as bi-templates with compressed CO_2_.

The possible formation mechanism of the PMOs with compressed CO_2_ have been investigated and explained in previous paper^61^. The result indicates that the acid caused by dissolving CO_2_ into water acts as catalyst in the hydration of organosilica precursors to form PMOs nanomaterials. Meanwhile, with the increase of pressure, CO_2_ can penetrate into the hydrocarbon-chain region of the sphere micelles to expand the volume region occupied by hydrocarbon-chain, causing the curvature decrease of the interfacial films, and the packing parameter becomes larger with the increasing of the bending energy. It is well known that the CTAB/SDS mixture surfactant systems have strong electrostatic interactions so that they tend to form a spherical/worm-like micelles or vesicle structure at an appropriate cationic/anionic ratio^67^. Spherical micelles were formed in the mixed surfactants system when the molar ratio of surfactants (CTAB/SDS)/P123 and BTEB was equal to 0.004:1 in the study. When the CO_2_ pressure increased from 3.90 to 4.90 MPa, the sphere micelles tended to aggregate and form the worm micelles due to the insertion of CO_2_ into the hydrocarbon-chain region. As the result, with the pressure increasing from 3.90 MPa (Fig. 1b) to 4.90 (Fig. 1c) and 5.90 MPa (Fig. 1d), the PMOs nanomaterials synthesized with CTAB/SDS as the soft template turned from spherical to worm-like nanoparticles. Moreover, block copolymers can absorb onto the CTA^+^-DS^−^ hydrophobic layer via the PPO blocks at a correct hydrophilic/hydrophobic balance to reduce the bending elasticity of the CTA^+^-DS^−^ hydrophobic layer. Thus, the P123 copolymers anchor on the micelles to serve as the template of mesoporous silica. Meanwhile, the micelles act as a “nucleus” to promote the further growth of these P123-templated mesoporous materials around the aggregation. The possible mechanism of the syntheses of PMOs with tubular structure using compressed CO_2_ was illustrated in Fig. 8. When the molar ratio of the surfactant and organosilane precursors increases from 0.004:1 to 0.017:1 (increase of the concentration of CTAB/SDS), the aggregation in the solution would translate from micelles to the vesicles. Just as shown in Fig. 7b, the worm-like nanoparticles and hollow nanospheres are obtained together during the synthesis. And with the molar ratio increases to 0.068:1, micelles in the solution have translated entirely to vesicles and thus only hollow nanospheres present in the prepared PMOs materials (Fig. 7c). Therefore, the variation of the morphology and structure of the obtained PMOs nanomaterials could be simple realized by adjusting the compressed CO_2_ pressure in the aqueous solution.

## Conclusions

In summary, PMOs nanospheres with tubular structure were successfully synthesized through a facile and green approach using compressed CO_2_ to replace acid or any base in the solution. This simple strategy was carried out with a bi-temple surfactant system where the successively grown PMOs nanospheres with tubular structure could be appropriately adjusted by manipulating the pressure of compressed CO_2_. Moreover, the pore size, pore volume and surface area of prepared PMOs nanospheres increased monotonically with the increase of CO_2_ pressure. The obtained PMOs nanospheres prepared with different pressures possessed a wide surface area (477–815 m^2^/g), adjustable pore volume (0.68–1.11 cm^3^/g), and uniform pore diameter (5.7–6.6 nm). Solid-state NMR and fourier transform infrared further affirm the existence of the organic group in the obtained nanomaterials. Considering the accessible tubular structure of PMOs nanospheres, the adsorbent and drug delivery system were constructed by loading and releasing of RB and IBU to show its remarkable adsorption and desorption ability of the PMOs nanospheres. Furthermore, different concentrations of surfactants were investigated and PMOs nanoparticles with different structure were obtained. The possible mechanism was due to the acid caused by dissolving CO_2_ into water can serve as catalyst in the hydration of organosilica precursors to form PMOs nanomaterials. Meanwhile, CO_2_ can penetrate into the hydrocarbon-chain region of the sphere micelles to expand the volume region occupied by hydrocarbon-chain, causing the packing parameter becomes larger with the aggregation transformed from micelle to vesicle.

## Experimental Details

### Materials

CO_2_ (>99.95%) was provided by Beijing Analysis Instrument Factory. Hexadecyl trimethyl ammonium bromide (CTAB), sodium dodecyl sulfate (SDS), absolute ethanol (99.98%), and hydrochloric acid (98%) were obtained from Sinopharm Chemical Reagent Co., Ltd. (Shanghai, China). 1,4-Bis(triethoxysilyl)benzene (BTEB, 96%), and Rhodamine B (RB) were purchased from J&K Chemical and Shanghai Chemical Reagent Factory (Shanghai, China), respectively. Ibuprofen (IBU) and triblock copolymer EO_20_PO_70_EO_20_ (Pluronic P123, 96%) were purchased from Sigma-Aldrich. All the reagents were used without further purification and the solutions were prepared with deionized water.

### Syntheses of PMOs nanospheres with tubular structure

CTAB/SDS and Pluronic P123 were used as soft bi-templates, and BTEB was used as the bridged organosilica precursor. In a typical synthesis of PMOs nanospheres, 0.1125 g CTAB and 0.0714 g SDS were dissolved into 7.50 g distilled water respectively. Then 0.0525 g P123 completely dissolved in 15.0 g distilled water was added to the CTAB/SDS solution to form a mixture of surfactants. After addition of 0.2142 g BTEB which was used as a precursor to produce silicas for PMOs nanospheres (molar ratio of the surfactants and BTEB is 0.004:1), the surfactants mixtures were transferred into a stainless steel autoclave under vigorous stirring (1200 rpm) and a certain amount of compressed CO_2_ was charged into the autoclave to contact with the reaction mixture. After stirred at 40 °C with a certain pressure of compressed CO_2_ for 24 h, the solution was heated to 100 °C and kept for another 48 h under static conditions. Meanwhile, the pressure was kept constant during the hydrothermal treatment processing. The solid product was collected by filtration and dried in automatic thermostat at 60 °C overnight, following by an extraction with 120 ml of 100:3 volume ratio of ethanol and HCl mixture using a Soxhlet apparatus for 48 h to remove the surfactant. Then the final product was dried in automatic thermostat at about 60 °C.

### PMOs nanospheres characterization

The transmission electron microscopy (TEM) images were taken on FEI Tecnai Spirit microscope operated at an accelerating voltage of 150 kV. The samples were sonicated for 30 min in an adequate quantity of ethanol, and the solution was dropped onto a porous carbon film on a copper grid and then dried. The porosity properties were gained from N_2_ adsorption-desorption isotherms using a Micromeritics ASAP 2020 M system. Solid-state magic-angle spinning (MAS) nuclear magnetic resonance (NMR) spectra were collected on a Bruker DRX400 MHz FT-NMR spectrometer with a MAS speed of 8 kHz. Cross-polarization (CP) technique was used for both ^13^C and ^29^Si spectra, which were referenced to tetramethylsilane. The fourier transform infrared (FTIR) spectrum of the prepared PMOs hollow spheres were recorded on a Bruker-Vector 22 FTIR spectrometer, and the samples were prepared by the KBr pellet method.

### Adsorption of RB

20 mg PMOs nanospheres were suspended in RB solution (10 mL) with concentration of 10, 20, 50, 100, 500 and 1000 mg/L at room temperature respectively. After slowly stirring the mixtures for 24 h at 100 rpm, the supernatants were separated out by centrifuge at 11,000 rpm for 10 min. Then the supernatants were diluted to an appropriate range for measuring its absorbency on UV/visible spectrophotometer (Persee TU-1800) at a wavelength of 553 nm, the concentration of the RB solution was subsequently obtained according to the Beer-Lambert Law. The Adsorption amount of RB was calculated by the difference in concentration of the RB solution before and after adsorption.

### IBU adsorption and release

20 mg PMOs nanospheres were degassed at 120 °C for about 12 h before suspended in 10 mL IBU/hexane (10 mg/mL) mixture solution. Then the mixture was sonicated for 30 min to make IBU dissolve adequately. After slowly stirring the mixtures for 36 h at 100 rpm, the supernatants were separated out by centrifuge at 11,000 rpm for 10 min. The solution concentration was determined by using UV-VIS spectrophotometer (Persee TU-1800) at 271 nm and the solid sample were washed with deionized water and dried. For release, typically, the obtained solid sample was added into 40 mL PBS buffer solution (pH 7.4), then agitated in a water shaker at 80 rpm, and the temperature was kept at 28 °C. The release profiles of IBU were determined by collecting 1 mL of sample solution from the mixture at different time. The IBU concentration in the sample solution was calculated with the same method as above.

### Syntheses of PMOs nanospheres with different molar ratios of the surfactants and organosilica precursor

In a typical synthesis, which is similar to the synthesis of PMOs nanospheres with tubular structure, except the ratio of the surfactants (CTAB/SDS)/P123 and BTEB were different. The amount of BTEB was fixed on 0.2142 g, with the molar ratio of surfactants and BTEB changed from 0.004:1 to 0.017:1 and 0.068:1, and the CO_2_ pressure is 4.90 MPa.

## Additional Information

**How to cite this article**: Huang, X. *et al*. A facile template route to periodic mesoporous organosilicas nanospheres with tubular structure by using compressed CO_2_. *Sci. Rep.* **7**, 45055; doi: 10.1038/srep45055 (2017).

**Publisher's note:** Springer Nature remains neutral with regard to jurisdictional claims in published maps and institutional affiliations.

## Supplementary Material

Supplementary Information

## Figures and Tables

**Table 1:** Structure properties of the PMOs nanospheres with tubular structure from nitrogen sorption measurements at different pressures.

Pressure (MPa)	BET surface area (m^2^/g)	Pore volume (cm^3^/g)	Pore diameter (nm)
3.90	477	0.68	5.7
4.90	781	0.97	6.4
5.90	815	1.11	6.6
