# Supplementary material for: A facile template route to periodic mesoporous organosilicas nanospheres with tubular structure by using compressed CO2
Source: Sci Rep. 2017 Mar 20;7:45055. doi: 10.1038/srep45055 (PMC5357914; doi:10.1038/srep45055)
Supplement: Supplementary Information [file srep45055-s1.pdf]

# A facile template route to periodic mesoporous organosilicas nanospheres with tubular structure by using compressed $CO_2$

*Xin Huang, Wei Li, * Meijin Wang, Xiuniang Tan, Qian Wang, Cheng Wang,*

*Mengnan Zhang, and Jing Yuan**

Department of Chemistry, Capital Normal University, Beijing, 100048 China. Tel:

+86-10-68903086, E-mail: wli@cnu.edu.cn; lilacjing@126.com

# Supporting Information

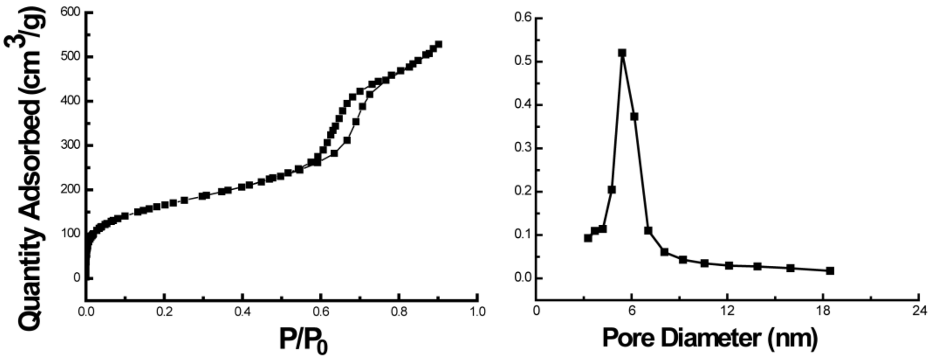


**Figure S1.** Nitrogen adsorption–desorption isotherm and pore size distribution of the PMOs nanospheres after loading RB.

**Table S1**. Structure properties of the PMOs nanospheres synthesized at 4.90 MPa before and after loading RB from nitrogen sorption measurements

| Samples | BET surface area ($m^2/g$) | Pore volume ($cm^3/g$) | Pore diameter (nm) |
|---|---|---|---|
| Before loading | 781 | 0.97 | 6.4 |
| After loading | 601 | 0.71 | 5.4 |
